# Impact of Corrosion on the Bond Strength between Concrete and Rebar: A Systematic Review

**DOI:** 10.3390/ma15197016

**Published:** 2022-10-10

**Authors:** Amadou Sakhir Syll, Toshiyuki Kanakubo

**Affiliations:** 1 Graduate School of Systems and Information Engineering, University of Tsukuba, Tsukuba 305-8577, Japan; 2 Division of Engineering Mechanics and Energy, University of Tsukuba, Tsukuba 305-8577, Japan

**Keywords:** bond strength, corrosion of steel bar, concrete

## Abstract

Corrosion of the reinforcement affects more than the cross-sectional area of the rebar. The volume of steel also increases due to expansive corrosion products, leading to the cracking, delamination, and spalling of concrete. As a result, the bond capacity between concrete and rebar is affected. Researchers have extensively examined the impact of corrosion on the bond strength between concrete and rebar to propose empirical, theoretical, or numerical predictive models. Therefore, research programs on this topic have increased rapidly in recent years. This article presents a systematic literature review to explore experimental methods, outcomes, and trends on this topic. The Web of Science search collected 84 relevant research articles through a rigorous selection. Key factors that affect bond strength degradation, including concrete cover, concrete strength, and stirrups, have been documented. However, a general model is still unavailable due to discrepancies caused by differences in testing methods to evaluate the effect of corrosion on bond strength. Furthermore, researchers attempted to clarify the degradation mechanism of bond strength affected by corrosion. As a result, new alternatives have been proposed to build a practical model to assess the bond strength deterioration of corroded structures.

## 1. Introduction

**Figure 1:**
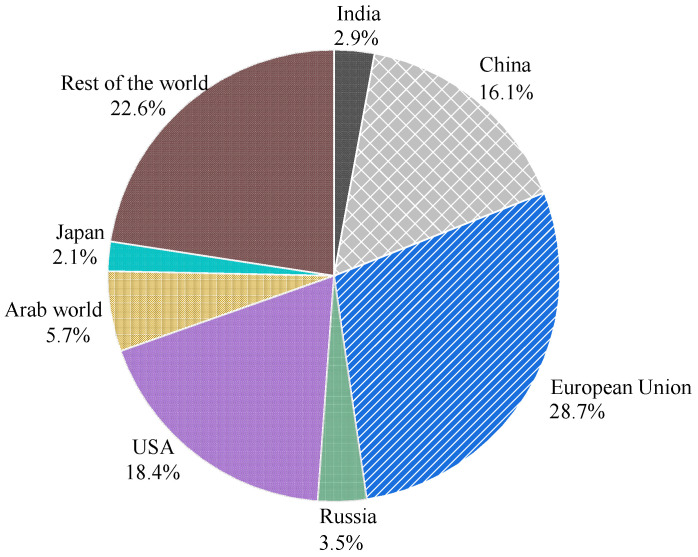
Economic loss due to corrosion.

In recent decades, corrosion has been identified as the most common cause that threatens the durability of reinforced concrete (RC). In recent decades, corrosion has been identified as the most common cause that threatens the durability of reinforced concrete (RC) under an aggressive environment. This generates enormous direct and indirect expenses [1,2]. The National Association of Corrosion Engineers (NACE) [3] reported that corrosion causes an annual loss of USD 2.5 trillion worldwide. Figure 1 shows the annual loss share in different countries.

The researchers demonstrated that the bond is more vulnerable to corrosion. Auyeung et al. [4] confirmed that the bond strength degradation of an unconfined concrete specimen with a corroded steel reinforcing bar (rebar) is much more critical than the cross-section loss. According to their findings, a diameter reduction of 2% could result in a bond loss of 80%. The investigation by Li and Zheng [5] also reveals that the structural degradation of the bond varies more than the loss in stiffness and strength. Thus, corrosion-induced bond deterioration becomes highly topical. However, the bond mechanisms between rebar and concrete are quite complex due to many influential factors. The perplexity may increase when the impact of corrosion on the bond is also investigated. Although researchers have extensively studied this subject, several knowledge gaps remain. In addition, the proposed models for bond strength degradation due to rebar corrosion are dispersed in the literature.

Some authors have also published review articles on this topic due to the rapid growth of the literature [6,7,8]. Lundgren [6] (2007) systematically described the effect of corrosion on the bond between reinforcement and concrete. Finite element analyses provided a basic understanding of the different cases. The FEM results are then compared with studies of experimental work to provide an overview of the influence of the rebar type, presence of stirrups, and confinement of concrete. Mancini and Tondolo [4] (2014) reviewed studies on corrosion-related bond degradation in RC members. The effects of bond length combined with various methods of the bond test were summarized in the study. In 2019, Lin et al. [8] reviewed the latest research on the bond deterioration of corroded rebars under cyclic or monotonic loads. The review described the most influential factors affecting corrosion-related bond degradation. Furthermore, various proposed models are compared to assess the bond behavior (bond strength and stress-slip curve). They highlighted that the degradation models are mainly based on specific test results and are not yet generalized.

This article aims to identify the most common methods, outcomes, and trends on the effect of corrosion on bond strength through a systematic literature review. The paper includes seven sections structured as follows. Section 2 presents the methodology for conducting the systematic review of the literature. Section 3 summarizes the experimental methods used to determine the bond strength in corroded specimens. In Section 4, the influential factors that affect the degradation of bond strength due to corrosion are analyzed. Section 5 examines empirical models to assess bond degradation. The challenge and new trends are reviewed in Section 6. Finally, the conclusion and limitations of this study are presented in Section 7.

## 2. Research Methodology

According to Palmatier et al. [9], a systematic review can help researchers to analyze the status of their field of study to reach clear conclusions. In the literature, several approaches and methods to adequately perform a systematic review are described in detail [10,11,12,13]. This study follows the guidelines described by Denyer and Tranfield [10]. The evaluation was carried out in five steps: (1) selection of objectives; (2) selection of databases; (3) identification of keywords; (4) selection of compatible papers; and (5) extraction of data. This approach has been chosen to make the process transparent and replicable. However, this article identifies methods, issues, and trends in corrosion-related bond strength deterioration. The methodology used to collect, analyze, and report the data is detailed in this article.

### 2.1. Search Strategy

**Figure 2:**
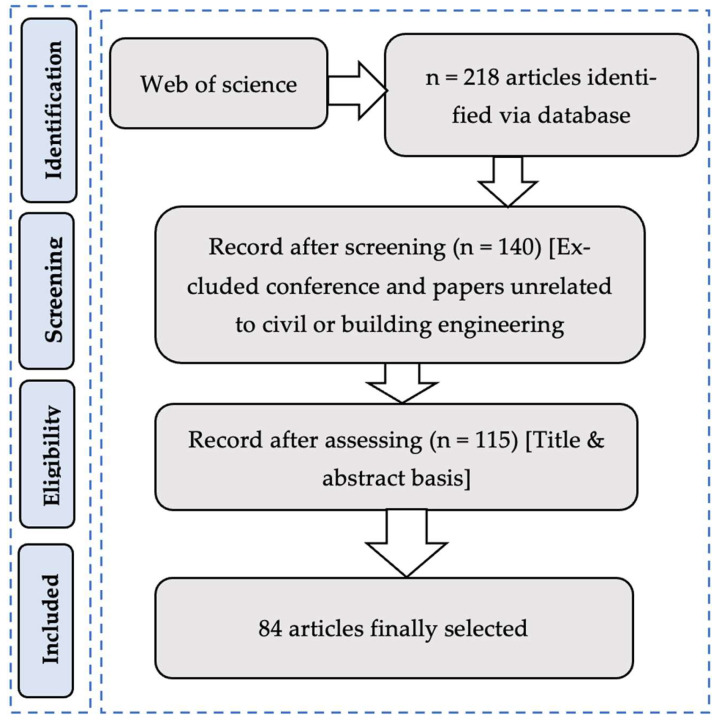
Research methodology.

Figure 2 summarizes the research methodology. The literature search was conducted using the “Web of Science” database due to its reputation for high-quality indexing publications in civil engineering [14]. In the Web of Science search, the research criteria were “title and keywords.” The search link can be found here [15]. To filter the literature, we formulated the query using the combination of keywords “Bond”, “concrete,” and “corro*.” The first search resulted in 218 articles. The number of articles was reduced to 175 after eliminating manuscripts that had not been peer-reviewed (letters, conference abstracts, and patents). The articles were then reduced to 139, including only those related to civil engineering, construction, or building applications. Finally, the title and abstract of the manuscript were manually selected to assess the relevant articles. In this step, the articles were selected if they included an experimental protocol and a bond strength assessment. Finally, 84 articles were considered in this review.

### 2.2. Search Result

**Figure 3:**
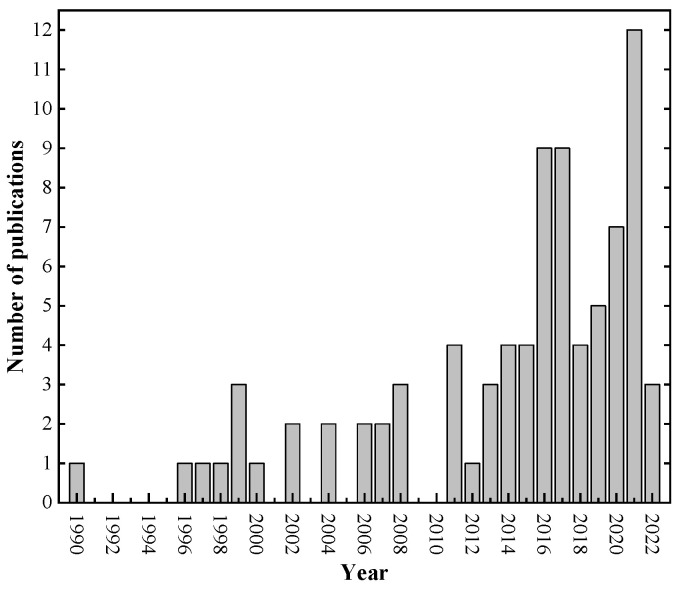
Time distribution of publications.

The study included 84 published articles from 1990 to 2022 (April). Scientific attention to empirical evaluation of bond strength in corroded reinforced structures has continuously grown over the past few years. Figure 3 shows that from 1990 to 2013, studies on this topic did not exceed four articles per year. From 2014, the number of publications increased, reaching six articles published in 2016 and 2017. However, the most significant increase was in 2021, when twelve works were published.

**Figure 4:**
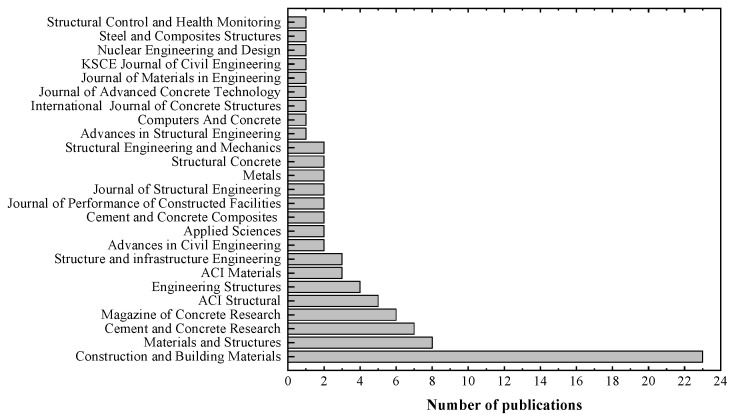
Distribution of articles by year of publication.

Figure 4 shows the sources of the selected publications. The journal “Construction and Building Materials” (22 articles) was the most prolific. Only four journals published more than four articles on the topic. Among them, “Materials and Structures” published eight articles, “Magazine of Concrete Research” published six articles, followed by “Cement and Concrete Research,” and “ACI Structural Journal” with five articles each. Moreover, four articles were published in “Engineering Structures,” and one to three articles were published in the remaining nineteen journals. It is interesting to note that most journals have a sectorial scope on construction materials. Only two journals (“Metals” and “Applied Sciences”) have a broad focus and publish articles unrelated to the field of civil engineering or building and construction.

**Figure 5:**
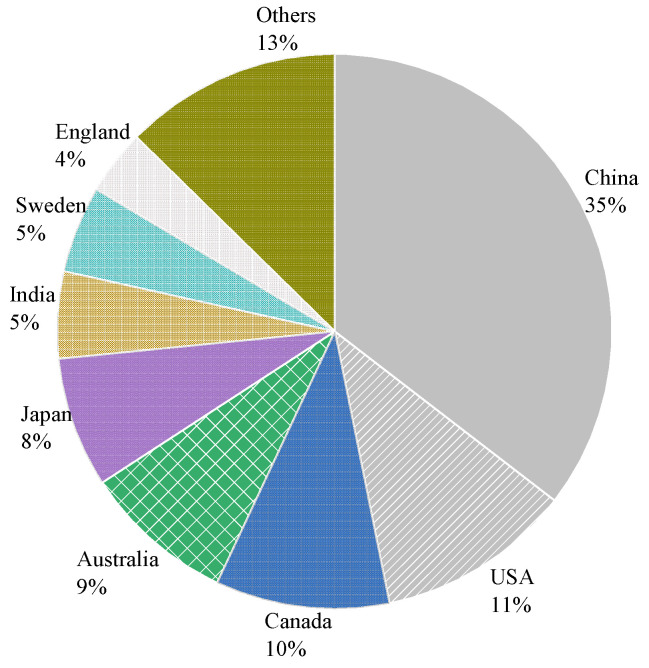
Geographical distribution of publications.

Furthermore, Figure 5 shows the geographical distribution of the authors of the selected articles. In terms of publications, China is the most influential (35%), followed by the United States (11%) and Canada (9%). These three countries have published up to half of the total publications. Although geographical distribution cannot provide a solid conclusion, this could help researchers develop collaboration, create joint venture studies, and exchange innovative technologies and ideas.

## 3. Test Specimens and Bond Test Methods

### 3.1. Corroded Bond Specimens

#### 3.1.1. Specimens from Decommissioned Structures

The most prominent and realistic conditions are obtained when using naturally deteriorated specimens to investigate the influence of corrosion. The authors [16,17,18,19] studied the effect of corrosion on the end anchorage by performing a four-point bending test on decommissioned edge beams from Stallbacka (Sweden). However, testing specimens from a decommissioned structure can be challenging because the influential factors are likely uncontrollable. Thus, the data can only be informative. In a recent article, Lundgren et al. [20] suggested an approach for selecting and designing bond tests using specimens from decommissioned structures.

#### 3.1.2. Accelerated Corrosion Method

**Figure 6:**
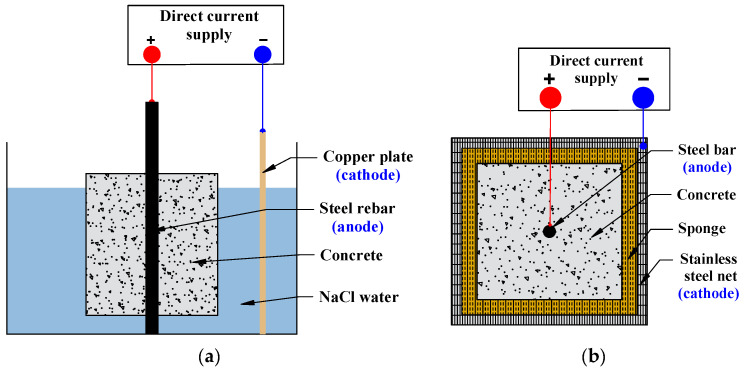
Accelerated electrical method: (**a**) Immersed specimen; (**b**) Wrapped specimen.

Natural corrosion is a relatively slow phenomenon. To reproduce this phenomenon in the laboratory, accelerated corrosion methods are commonly used, including dry-wet cycles, salt spray tests, and accelerated electrical corrosion. The electric accelerated corrosion test is frequently used to study the bond performance of corroded RC because of its benefits. (short time, controllable current density, and portable test equipment) [21]. The approach is primarily electrochemical in nature, with rebar serving as the anode and stainless steel or copper plate serving as the cathode. The corrosion of the rebar is then accelerated by applying a direct current. To ensure the normal conduct of the electric accelerated corrosion test, approximately 5% sodium chloride is often added to fresh concrete. The specimen is either immersed in a salt solution or wrapped with a humidified sponge, as illustrated in Figure 6.

### 3.2. Experimental Bond Test Setup

#### 3.2.1. Pull-Out Test

**Figure 7:**
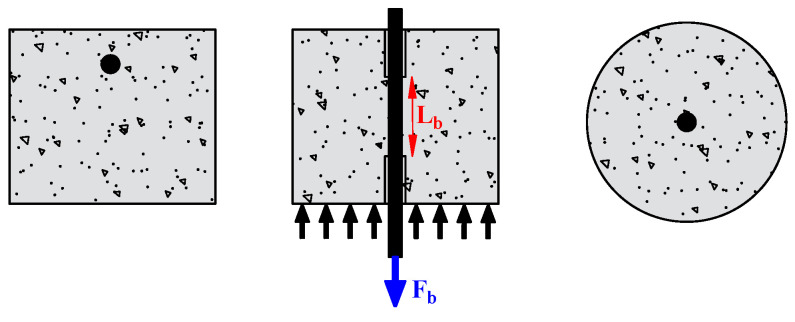
Pull-out test.

**Figure 8:**
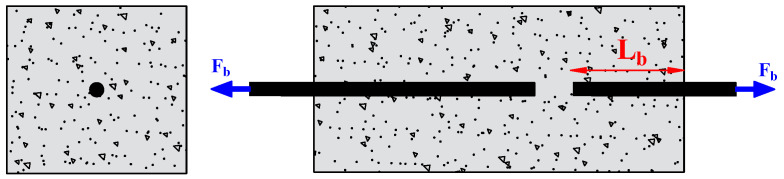
Pull-out test modified by Auyeung.

The researchers used the pull-out test to measure the bond strength because of its simplicity and high replicability. It consists of applying a tensile force to pull-out steel bars embedded in the concrete, as shown in Figure 7. A short bond length (mostly five times the diameter of the rebar) is generally adopted to focus on the local bond behavior. Depending on the position of the main tested rebar, the pull-out test can be referred to as central or eccentric. However, in the pull-out test, the rebar is in tension, and the concrete is in compression, which does not reflect the actual situation in the structure. To overcome this drawback, Auyeung et al. [4] used a modified version of the concentric pull-out test. They set two aligned rebars with different embedding lengths. One end of the longer rebar is fixed, and the shorter embedding length is pulled out to determine the bond strength (Figure 8).

#### 3.2.2. Beam Test

**Figure 9:**
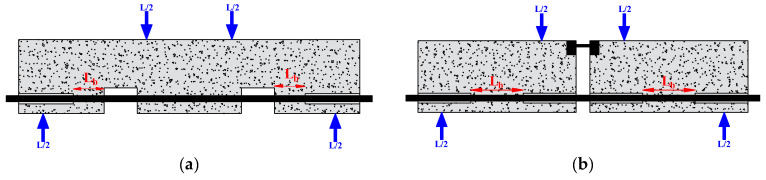
Beam test: (**a**) with a pocket; (**b**) with a hinge.

**Figure 10:**
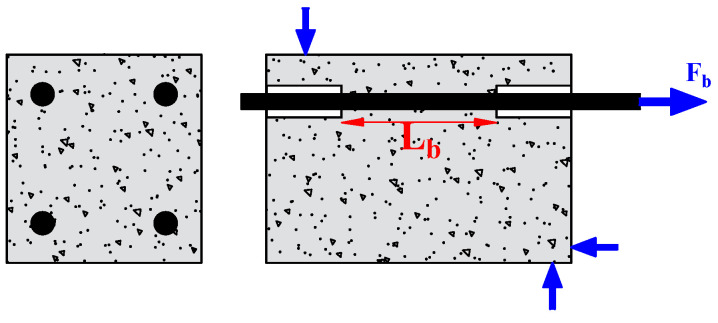
Beam end test proposed by Chana [28].

Some authors also evaluated the bond behavior using a beam in four-point bending to replicate the actual stress in RC beams [18,22,23,24,25]. Bond performance is measured using the steel bars arranged in the tension area, as shown in Figure 8. Mangat and Elgarf [26] used another variant of a hinged beam (Figure 9b). Compared to the pull-out test, the beam test is more realistic, including the bending moment and shear in the RC member [27]. In addition, the bond is investigated while both the concrete and the rebar are under tension. However, the beam test is generally less widely used due to its complex operation, high cost, and relatively low replicability. Therefore, Chana [28] designed a beam end specimen with parallel bars cast around the four corners. As a result, the bond strength can be tested for both top and bottom cast conditions. (Figure 10).

**Figure 11:**
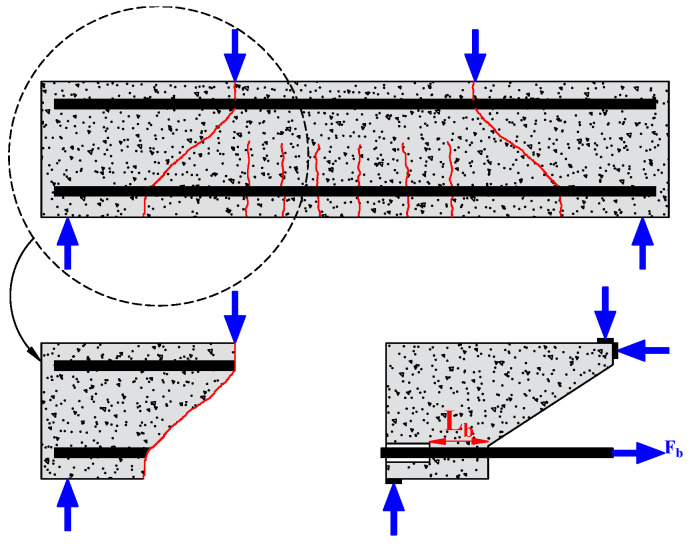
Modified Beam test by Hanjari et al. [29].

Furthermore, Hanjari et al. [29] further simplified the beam test. As shown in Figure 11, only the supported part of the beam end was selected, and the reaction force of the part was simulated, which reduced the test cost and improved the repeatability.

The presence of corrosion increases the uncertainties, leading to a greater scattering of the bond strength. Each test method features some characteristics of bond behavior. Therefore, a unified standard for the bond test cannot be identified because any test cannot fully describe the bond behavior.

## 4. Bond Strength Deterioration Due to Corrosion

### 4.1. Corrosion of the Main Rebar

**Figure 12:**
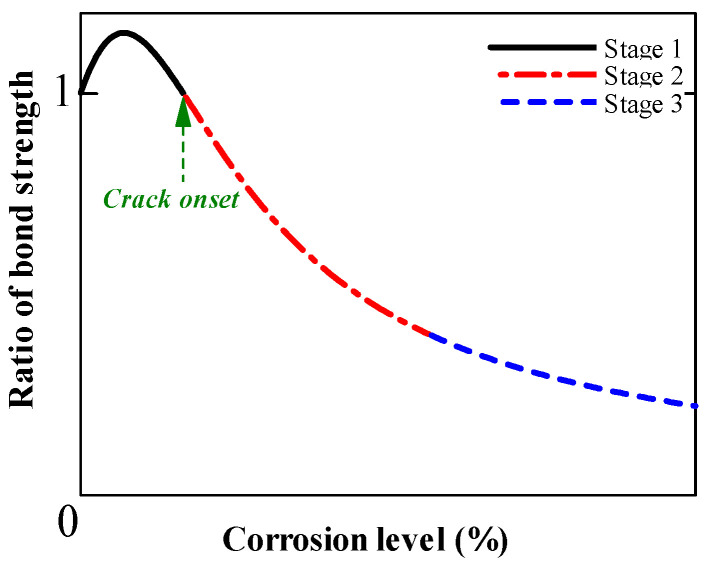
Degradation of bond strength with corrosion level.

Several investigations have examined the impact of longitudinal rebar corrosion on bond strength [30,31,32,33,34,35]. The findings point to a general trend of bond strength deterioration owing to corrosion. Figure 12 shows the bond strength ratio of corroded rebar to non-corroded rebar at various degrees of corrosion. Three stages characterize the degradation trend. In stage 1 (low levels of corrosion), the production of expansive corrosion products could improve confinement to the rebar, increasing the bond strength. Expansive materials can crack the concrete cover as corrosion develops, rapidly decreasing bond strength (stage 2). In stage 3 (high levels of corrosion), the bond strength did not alter with increasing mass loss, asymptotically approaching a limit. Significant rib deterioration would result in friction-type behavior comparable to plain bars [36,37,38,39]. As a result, the bond strength slowly decreases in stage 3.

### 4.2. Essential Factors Affecting the Bond Strength of Corroded Specimens

The selected articles revealed that several factors affect the bond strength deterioration, including the type of corrosion (uniform/ pitting, due to chloride or carbonation; wet or dry environment); the amount of stirrup; the position of the main bar; concrete cover; bar diameter and concrete strength. However, this review included only three factors (concrete strength, concrete cover-to-rebar diameter ratio, and stirrup). These factors were selected because the authors considered them to have the most significant influence. Moreover, the selected influencing factors are rather clearly definable.

#### 4.2.1. Influence of Concrete Strength

**Figure 13:**
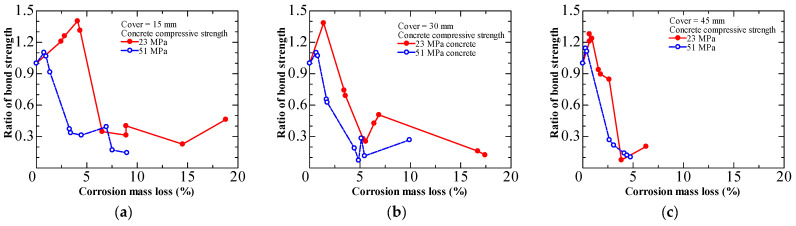
Influence of concrete strength on bond deterioration from Yalciner et al. [40]: (**a**) cover = 15 mm; (**b**) cover = 30 mm; (**c**) cover = 45 mm.

The bond performance of sound RC elements is proportional to the strength of the concrete. Increased concrete strength leads to increased bond strength. Yalciner et al. [40,41] investigated the effect of concrete strength on bond loss due to corrosion. They conducted a pull-out test on unconfined specimens with two different concrete strengths (23 MPa and 51 MPa). The results summarized in Figure 13 demonstrated that corroded specimens with higher concrete strength showed a more substantial bond strength degradation. This is probably because the brittleness of the corroded specimens caused an abrupt loss of bond strength. Corrosion products do not diffuse rapidly into concrete pores because of the excellent resistance to permeability in high-strength concrete. Therefore, the accumulation of expansive products around the rebar leads to more significant induced crack widths, leading to a more severe deterioration of the bond strength [42,43].

**Figure 14:**
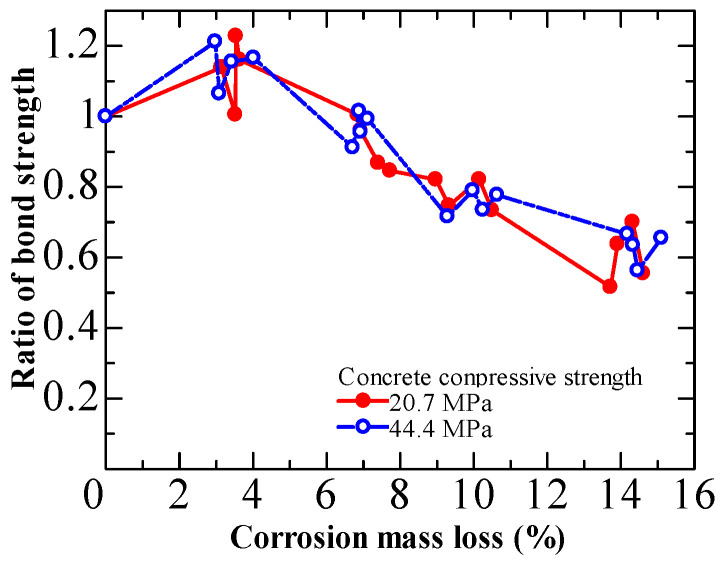
Influence of concrete strength on bond deterioration from Zhou et al. [42].

Figure 14 shows the results of Zhou et al. [44,45]. They examined the effect of concrete strength on bond degradation using two different concrete mixes (20.7 MPa and 44.4 MPa) in specimens with stirrups. In contrast, they discovered that the compressive strength of the concrete did not affect the bond degradation trend. They emphasized that the mixing of different failure modes can complicate the observation of the concrete strength effect.

#### 4.2.2. Influence of the Cover-to-Rebar Diameter Ratio c/d

The concrete cover thickness c to steel bar diameter d (c/d) ratio is frequently regarded as a crucial element influencing bond strength. The bond strength was observed to increase as c/d increased. However, this increase is limited; for example, the bond strength will remain stable when c/d ≥ 3 in the specimen without stirrups [46]. Al-Sulaimani et al. [31] performed a pull-out using corroded specimens where three cover-to-diameter ratios (c/d) ratios of 3.75, 5.36, and 7.5 were adopted over 20, 14, and 10-mm bars, respectively. Data showed that 4% of corrosion is needed to start cracking for a c/d ratio of 7; however, about 1% is necessary to crack specimens with a c/d ratio of 3. They suggested that the ratio of cover-to-diameter (c/d) could be considered a significant factor that expresses corrosion protection.

**Figure 15:**
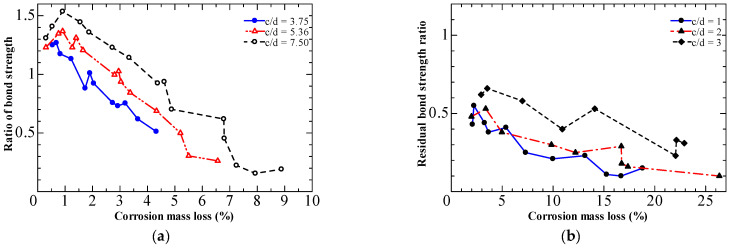
Influence of the cover-to-diameter ratio on bond deterioration: (**a**) Al-Sulaimani et al. [31]; (**b**) Amleh et al. [32].

Furthermore, Al-Sulaimani et al. [31] showed that bond deterioration is more severe in specimens with a smaller c/d, as shown in Figure 15a. The results of Amleh et al. [32] shown in Figure 15b are consistent with those of Al-Sulaimni et al. Concrete can still transfer stress across cracks. As a result of the residual confinement, the specimens with a more significant concrete cover demonstrated stronger bond strength.

#### 4.2.3. Influence of Stirrups

**Figure 16:**
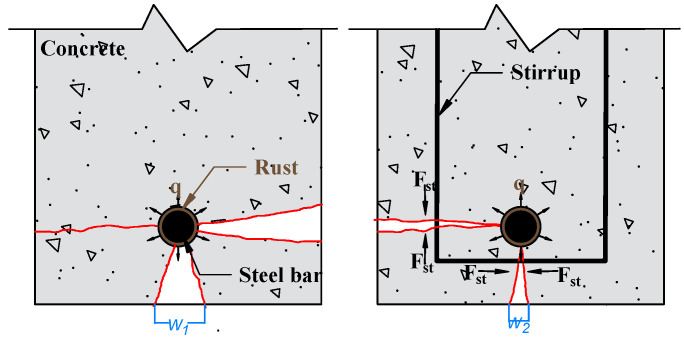
Effect of stirrups on corrosion-induced cover cracking. Reprinted with permission from [47]. 2016, Elsevier.

The influence of stirrups on the deterioration of the bond as a result of corrosion is twofold. Studies showed that the stirrup could increase concrete confinement, limiting the width of cracks due to corrosion [47,48,49,50,51], as illustrated in Figure 16. Second, several authors investigated the effect of stirrup corrosion on bond degradation. Fang et al. [38,39] performed a centric pull-out test on corroded specimens with and without stirrups. They discovered that a moderate corrosion rate (about 4%) had no significant influence on bond strength. However, bond degradation was observed when the degree of corrosion was greater than 6%. Zhou et al. [52] focused on the effect of corroded stirrups on the bond performance of reinforced concrete. They concluded that the bond strength improved when the degree of stirrup corrosion was less than 10%. However, bond degradation was observed when the degree of stirrup corrosion reached 15%. These findings are likely related to the corroded stirrup that produces hoop stress that acts inside and outside. This stress can crack the cover and add more confinement to the core concrete where the tested rebar is located [53,54,55]. As a result, the bond strength can increase.

Earlier studies have recognized that an adequate amount of stirrups can maintain the bond even in cracked concrete. However, stirrups often have a minor concrete cover and are the most vulnerable to corrosion. Furthermore, Hanjari et al. [29] concluded that considerable bond degradation occurs only when the stirrup corrosion is extremely high. However, some stirrup legs are fractured at pitting points or almost consumed by uniform corrosion in such extreme situations.

## 5. Modeling of Bond Strength Deterioration Due to Corrosion

Researchers have used different techniques to derive models to predict corrosion-related bond strength degradation. Most models are based on specific experimental results and consider different parameters. Furthermore, Wang et al. [56,57] and Bhargava et al. [58,59] used a thick-walled cylinder approach to propose a theoretical model, further validated by experimental results. Other authors also collected data from previous studies to propose bond degradation models using statistical analysis [60,61] or deep learning [62,63,64,65]. The major limitation of these models is that they are based on assumptions regarding the value of essential input factors that are not consistently measurable. In addition, the models go through a sequence of complicated integration and derivation techniques. In this reference [66], an extensive database of experimental studies on corrosion-related bond degradation is available.

The following subsections present some empirical models. The proposed model can be divided into two main categories: a model based on corrosion mass loss and a model based on induced crack width.

### 5.1. Models Based on Corrosion Mass Loss

**Figure 17:**
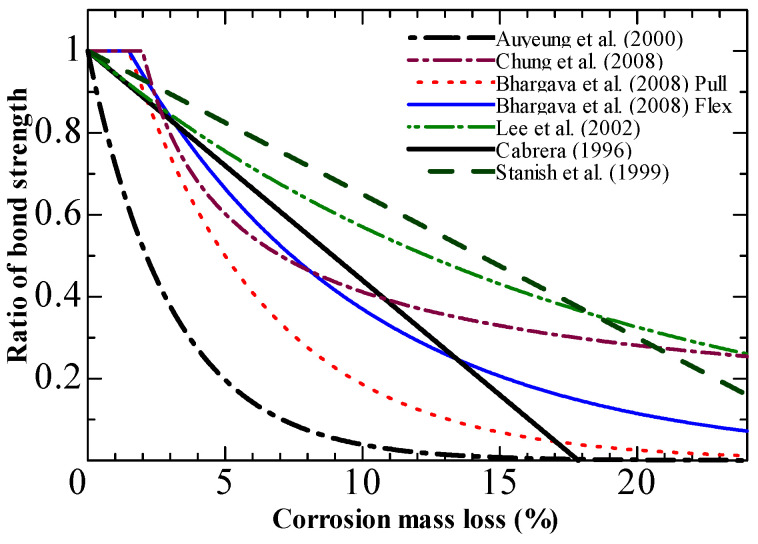
Comparisons of model predictions based on mass loss [4,42,67,68,69,70].

The suggested models [4,42,67,68,69,70] agree with the experimental data for which they were calibrated. Figure 17 indicates little agreement among the suggested models despite some degradation trend similarities.

The same degradation trends are observed in almost all proposed empirical models. Due to the limited amount of test data used for validation, each model claimed to be able to evaluate bond loss with reasonable accuracy. However, the models are characterized by their dispersion of the bond loss level. The scatter can be attributed to the specimen design or uncertainty related to the complex corrosion process.

However, it is found that this model, where mass loss is the main parameter, is likely challenging to implement. The mass loss of corroded rebar is not easily measurable in real situations. Therefore, to implement these models, engineers would first need a model to correlate the measurements of surface crack widths with the “hidden” internal corrosion. However, many uncertainties associated with each model can seriously weaken its effectiveness and accuracy.

### 5.2. Models Based on Induced Crack Width

Corrosion-induced surface cracking is easily measurable in actual structures and is convenient for practical use. Law et al. [71,72,73] investigated the effect of concrete cracking on bond deterioration. They claimed that bond strength correlates with induced crack widths rather than corrosion level. Furthermore, they underlined that the maximum crack width showed a stronger relationship than the average. In addition, the study by the same authors has highlighted the influence of the cover-to-diameter ratio on deterioration [71]. Their test data were better fitted with a linear or logarithmic function because corrosion has little influence on cracking when a certain crack width is reached.

Lin et al. [74] made major contributions by examining the relationship between surface crack widths and bond degradation. They used accelerated corrosion and eccentric pull-out experiments to examine the effects of various factors, such as bond length, concrete cover, corrosion level, and stirrup spacing. They proposed a mathematical model to assess bond loss using the surface crack width as the main parameter, as expressed in Equation (2).

(1) $$\tau_{u}\left(w_{ave},w_{stave}\right)=\tau_{u}\left(0\right)D_{st}(1.0-0.9e^{-20p_{st}}(1.0-e^{-1.73w_{ave}e^{-56.6p_{st}}}))$$

where $w_{ave}$ is the average longitudinal crack width; pst is the stirrup index, *pst* = *A_st_/CS_st_*; *A_st_* is the cross-sectional area of the stirrup; *S_st_* is the stirrup spacing, and *C* is the concrete cover. *D_st_* is a function of the average lateral crack width wstave.

(2) $$D_{st}=1-0.68(\frac{w_{stave}d_{st}}{-0.29C_{st}+1.58d_{st}}+1-(1-\frac{\theta }{d_{st}}(7.53+9.32\frac{C_{st}}{d_{st}})10^{-3}{)}^{2})$$

where *C_st_* is the concrete cover of stirrups; *d_st_* is the stirrup diameter; θ is the pit concentration factor.

**Figure 18:**
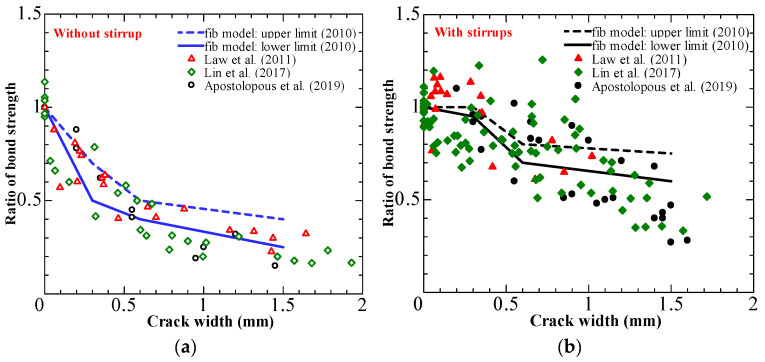
Comparisons of fib model [75] with test results in the literature [73,74,76]: (**a**) specimens without stirrups; (**b**) specimens with stirrups.

The fib model [75] provides a simplified relationship between corrosion-induced crack width and bond deterioration. Figure 18 compares experimental data from the literature [71,72,73,74,76,77,78] with the simplified correlation of the fib model [75].

Figure 17 indicates a strong correlation between induced crack widths and bond deterioration in unconfined specimens. Furthermore, fib model code estimations for specimens without stirrups are well predicted. The data scatter is higher for specimens with stirrups. The fib model fails to predict the increase in bond strength caused by stirrups, resulting in an overestimation.

## 6. Challenges and New Trends

The corrosion of rebar leads to a reduction in the steel cross-section, change in the concrete-rebar interfacial layer, and cracking of the concrete. These damages entirely affect the bond strength of the corroded specimen. However, these effects lead to difficulties in analyzing the processes at the fundamental level and negate the overall accuracy of the proposed models. Furthermore, a literature review showed that a unified model for general validity is not yet available. The discrepancies found in the data were mainly attributed to specimen variability, experimental machine setup, corrosion rate, or corrosion type (uniform or non-uniform). The following subsection summarizes the previous work to address this challenge. In addition, new alternatives for practical models are introduced.

### 6.1. Influence of the Corrosion Rate

Generally, researchers have widely adopted an accelerated corrosion setup with different current densities to replicate the natural corrosion effect relatively quickly. However, the densities of the accelerated corrosion current can be thousands of times higher than those measured under natural conditions [79,80]. Some authors focused on the influence of the induced current density on bond deterioration. An increase in current density has been shown to worsen bond deterioration [81,82]. Furthermore, Alonso et al. [83] concluded that current density reduces the effect of oxide formation on cracking, perhaps due to the production of oxides with a lower volumetric expansion ratio. Furthermore, Corronelli [84] pointed out that material degradation due to electrical current can worsen bond degradation when a high level of current density is applied.

**Figure 19:**
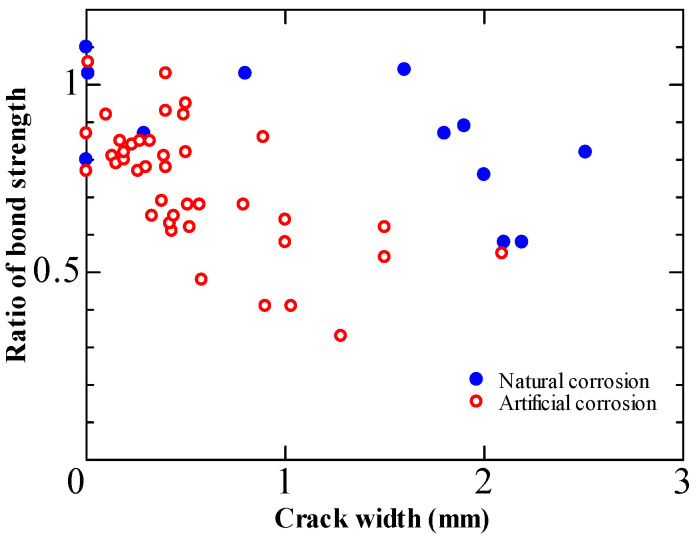
Influence of corrosion rate on the deterioration of bond strength [17].

On the other hand, researchers investigated the bond deterioration of corroded specimens extracted from decommissioned RC bridges [16,17,18,19]. Tahershamsi et al. [17] examined the bond degradation due to corrosion-induced crack widths of 32-year-old naturally corroded RC girders. They found more significant crack widths for given levels of corrosion than previous researchers using accelerated corrosion. Figure 19 shows that the reduction in bond strength in the naturally corroded specimen was less significant than the results of artificial corrosion. They attributed the disparity to the difference between natural and accelerated corrosion and the cumulative effects of freeze-thaw and corrosion. In conclusion, they stated that the estimation of bond deterioration using accelerated corrosion would be on the safe side.

Although a universally accepted current density limit does not yet exist, El Maaddawy and Soudki [85] recommended that current densities less than 200 μA/cm^2^ are preferable for more realistic data in accelerated tests.

### 6.2. Non-Uniformly Corroded Steel Bar

**Figure 20:**
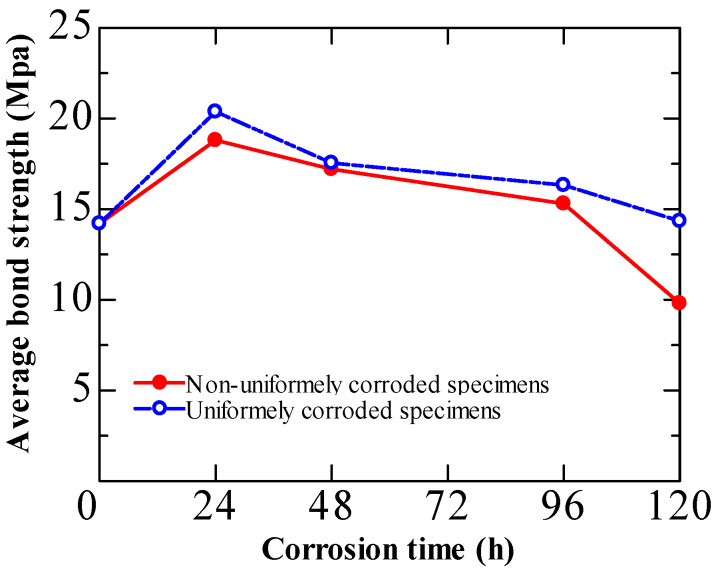
Influence of corrosion type on bond strength [87].

Previous investigations on the effect of corrosion on the bond strength of RC specimens were conducted by accelerated corrosion using impressed currents. As a result, the tested rebars were uniformly corroded. In this way, only mechanical interlocking and friction contributed to the bond strength of the corroded specimens. However, the natural corrosion of steel is typically non-uniform around the surface of the rebar [23]. Therefore, the interfaces of the corroded and non-corroded sections contribute to the bond. To address this, studies [86,87,88] focused on the influence of the rebar’s non-uniform corrosion on the bond strength’s deterioration. Fu et al. [87] adopted two corrosion modes for corroded RC specimens to induce non-uniform and uniform corrosion. Figure 20 shows that the bond strength degradation was generally more severe in non-uniformly corroded specimens than in the case of uniform corrosion. Deterioration becomes more noticeable as the corrosion level increases. This is likely related to the stress concentration within the interface.

### 6.3. Damage Identification of Bond in Corroded Specimens

**Figure 21:**
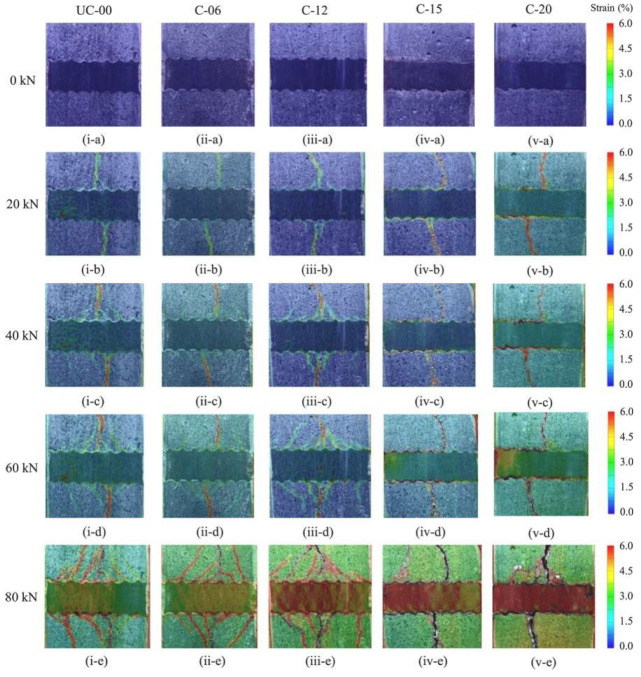
Change in strain distribution with loading. Reprinted with permission from [91]. 2021, Elsevier. Specimens UC-00, C-06, C-12, C-15, C-20 had degrees of corrosion of 0%, 6%, 12%, 15% and 20%, respectively.

Many factors affect the bond between rebar and concrete, leading to a complicated interaction. Recently, researchers have attempted to clarify damage using acoustic emission [89], ultrasonic technology [90], or digital image correlation (DIC) [91,92]. Ouglova et al. [87] used DIC to examine the beginning of bond failure in corroded specimens. They found that the increase in corrosion level slows the beginning of bond failure. Furthermore, the average bond stress at the start of the slippage is smaller than the bond strength during the pull-out test. Avadh et al. [91] used corroded specimens with a window to directly observe the rebar and concrete interface during the uniaxial tension test. The corroded rebars were cast in new concrete to eliminate the hindrance caused by the corrosion-induced cracks. They performed a DIC to examine the influence of rib height reduction and rust’s presence on bond failure. Figure 21 shows the change in strain distribution with loading adopted from Avadh et al. [86]. The onset and progression of diagonal cracks are observed in uncorroded specimens and specimens with degrees of corrosion up to 12% (Specimens UC-00, C-06, and C-12). Furthermore, failure-cracking observations revealed that the increased corrosion degree led to faster debonding between the rebar and the concrete. Diagonal cracks were not observed in specimens with higher degrees of corrosion despite having ribs (Specimens C-15 and C-20).

### 6.4. Clarification of the Effect of Corrosion on Bond

In the recent literature, researchers [93,94,95] attempted to investigate the separate effect of corrosion-induced cracks, corroded rebar shape, and rust around the rebar on the bond properties of reinforced concrete (RC) members. The results of Yang et al. [93] contribute to understanding the isolated effect of different corrosion damage on bond degradation. The first group included uncorroded specimens, and the specimens were subjected to accelerated corrosion in the second group. The specimens in the third and fourth groups were obtained by recasting the artificially corroded rebar into new concrete. The corroded rebars were cleaned for the third group (without rust) and intact for the fourth group (with rust). The authors concluded that corrosion-induced cracks were the primary cause of bond degradation.

Furthermore, its effect was more significant than the variations in the rebar profile or accumulation of rust at the interface. Jiradilok et al. [96,97] confirm Yang et al.’s [93] point that corrosion cracks have a dominant effect on bond behavior. Furthermore, they stated that the commonly used experimental method could not capture several factors due to corrosion. However, only the effect of surface cracking could be observed in the final result.

On the other hand, Mak et al. [94] changed the sealing condition of diverse specimens to vary the flow of rust through the concrete voids. They successfully separated the level of corrosion and the expansive effect that cracks the concrete. Their results showed it is challenging to correlate bond deterioration and rebar corrosion directly. Therefore, the width of the concrete cracks could be a better indicator than the corrosion level to assess the bond strength degradation.

### 6.5. Trend: Toward a Direct Crack-Based Model

Novel methods have been proposed to simulate cracking due to corrosion to overcome limitations related to electrical corrosion techniques. Crack width is used as a parameter to assess bond deterioration discretely. This alternative approach is based on previous findings demonstrating that the bond mechanism through interlocking ribs predominates over friction after cracking. Furthermore, it is assumed that a direct relationship between interlock reduction and crack width ignores the ambiguity related to corrosion product accumulation.

#### 6.5.1. Cracking Induced by a Splitting Load

Desnerck et al. [98] conducted a pull-out test on cracked specimens to focus on the more fundamental effect of the induced cracks. Before loading, the specimens were subjected to a split cylinder test to induce cracks. Two-line loads are applied to the specimen on opposite sides and along the concrete cylinder’s axis until the concrete’s first cracking. At this point, the specimen is unloaded. Their results showed that the bond strength degradation for double-cracked specimens was 65% higher than for single-cracked specimens. Furthermore, the effect of crack inclination on the rib pattern is negligible. Mousavi et al. [99] also adopted the same method to induce cracks. Although research has shown an interesting result, this method is not easily replicable.

#### 6.5.2. Cracking Induced by Expansion Agent Filled Pipe

**Figure 22:**
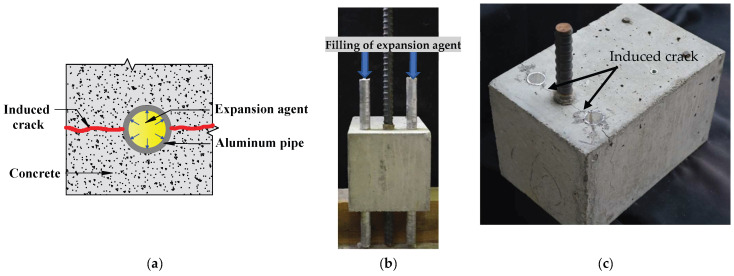
Cracking of concrete with EAFP: (**a**) cracking process; (**b**) filling with expansion; (**c**) example of cracking (adopted from Syll et al. [100]).

On the other hand, Syll et al. [100,101] have proposed a novel method to induce concrete cracking. An expansion agent is poured into an aluminum pipe embedded in concrete to simulate the expansion of the rebar volume due to corrosion, as shown in Figure 22a. The expansion agent is a non-explosive demolition agent mainly used to destroy rocks and reinforced concrete structures. In powder form, it expands when humified with 30% water. The specimen is positioned such that the aluminum pipe axis is vertically set to quickly pour the expansion agent, as shown in Figure 22b. The crack width increases over the time that has elapsed after filling. Thus, one can quickly obtain a target crack width by monitoring the time. The findings demonstrate that the nature of splitting cracks significantly affects bond strength degradation, with bond strength loss being more severe in a “side-split” than in a “single-split.”

**Figure 23:**
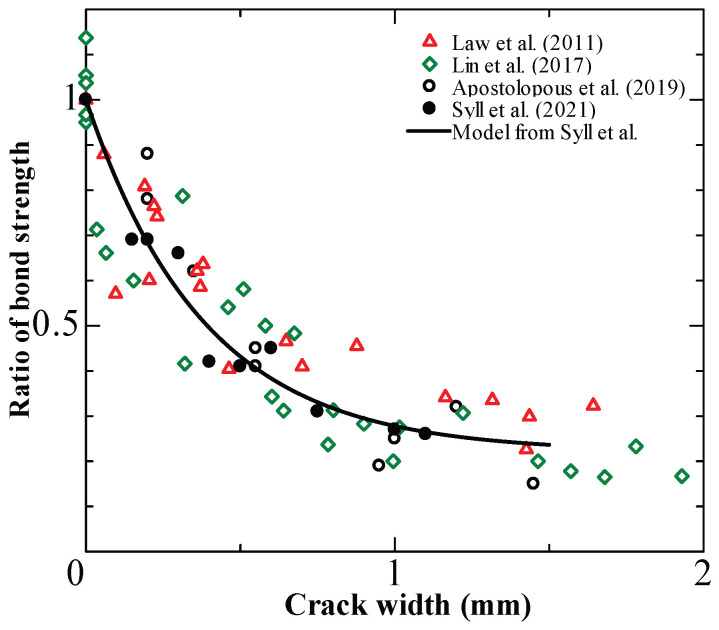
Comparison of Syll et al. [100] formulas with experimental results from the literature [73,74,76].

Furthermore, considering the induced crack width, an empirical model is provided to predict the reduction in bond strength due to rebar corrosion. Figure 23 shows that these prediction models correlate well with the available literature [73,74,76]. This confirms that the direct relationship between interlock reduction and crack width can ignore the ambiguity related to corrosion product accumulation. In conclusion, EAFP-induced cracks can effectively quantify the net damage due to corrosion. Because the induced crack width is the most obvious indicator of corrosion, it is convenient to express the degradation of the bond directly with this easy-to-measure damage indicator.

## 7. Conclusions and Limitations

The systematic review of the literature conducted in this work found that issues related to bond strength degradation due to corrosion have received significant attention in recent years. Furthermore, this article presents a general overview of the effect of corrosion on the bond strength between concrete and rebar. The following conclusions can be drawn:

1. The following variables are most frequently used in the literature to build models that predict the bond strength of corroded RC elements: corrosion mass loss, corrosion-induced crack width, stirrup quantity, and ratio of cover to bar diameter c/d.
2. The confinement provided by stirrups and concrete cover is essential to limit the deterioration of the bond due to corrosion. However, the influence of the concrete strength on bond deterioration remains unclear.
3. Most available bond strength degradation models based on corrosion mass loss do not adequately account for the contributing factors. Moreover, they are inadequate for practical use. Models using surface crack width as the governing parameter perform better; however, they can still be improved.
4. Most data were obtained using different current densities with various bond test setups on artificially corroded specimens. Therefore, a general model is still unavailable due to discrepancies caused by differences in testing methods to evaluate the effect of corrosion on bond strength.
5. In recent literature, the authors effortlessly attempted to clarify the mechanism of bond strength degradation due to corrosion. As a result, new alternatives have been proposed to build a practical model to assess the deterioration of the bond strength in corroded structures. Indeed, researchers should harmonize their efforts between different research programs to achieve consistent results in this field.

The following are some possibly fundamental parameters for future research:

1. Concrete properties such as strength and porosity.
2. Confinement (stirrup, concrete cover, or lateral pressure) affecting the bond strength before and after cracking.
3. Effect of smooth rebar bond strength degradation (rebar mainly used in old RC structures).

Since the search for articles was limited to peer-reviewed articles in English indexed on the Web of Science, the study findings do not entirely reflect the available literature. Future supplementary data could be collected by searching publications from various databases for quantitative and qualitative studies (e.g., Google Scholar, Scopus, Science Direct). Second, the focus of this study was limited to experimental studies of the effect of corrosion on bond strength only. Further research might explore the literature on bond-slip relationships or numerical methods to evaluate the consequences of corrosion on the bond.

## Data Availability

Not applicable.
